# Follow-up imaging after cryoablation of clear cell renal cell carcinoma is feasible using single photon emission computed tomography with ^111^In-girentuximab

**DOI:** 10.1007/s00259-019-04613-z

**Published:** 2019-11-25

**Authors:** Tim J. van Oostenbrugge, Johan F. Langenhuijsen, Egbert Oosterwijk, Otto C. Boerman, Sjoerd F. Jenniskens, Wim J. G. Oyen, Jurgen J. Fütterer, Peter F. A. Mulders

**Affiliations:** 1 Department of Urology, Radboud University Medical Center, 6500 HB Nijmegen, the Netherlands; 2 Department of Radiology and Nuclear Medicine, Radboud University Medical Center, Nijmegen, the Netherlands; 3 Department of Radiology and Nuclear Medicine, Rijnstate Hospital, Arnhem, the Netherlands

**Keywords:** CAIX, Cryoablation, Follow-up, Renal cell carcinoma, Single photon emission computed tomography

## Abstract

**Purpose:**

Detection of residual or recurrent vital renal tumor on follow-up (FU) cross-sectional imaging after ablative therapy is challenging. The specific and high expression levels of carbonic anhydrase IX (CAIX) in clear cell renal cell carcinoma (ccRCC) makes it a suitable target for imaging using radiolabeled anti-CAIX antibody girentuximab. The objective of this study was to evaluate the feasibility of targeted FU imaging 1 month after cryoablation of ccRCC using single photon emission computed tomography (SPECT) after ^111^In-labeled girentuximab administration.

**Methods:**

In this prospective study 16 patients underwent ^111^In-girentuximab-SPECT before MR-guided renal cryoablation between February 2015 and September 2018. In case of tumor targeting ^111^In-girentuximab-SPECT was repeated 1 month following MR-guided cryoablation. Presence of residual or recurrent vital tumor was assessed on contrast-enhanced cross-sectional imaging during further FU. The standard FU imaging protocol consisted of MRI/CT scans at 1, 3, 6, 12, and 18 months and annually thereafter.

**Results:**

A total of 10 (63%) patients showed positive tumor targeting on ^111^In-girentuximab-SPECT before cryoablation and 9 ( 56%) were eligible to undergo FU SPECT. Of the 9 ^111^In-girentuximab-SPECT FU scans, 8 (89%) were considered negative. One (11%) scan showed uptake suggestive for residual vital tumor. Six months after treatment, FU CT showed contrast enhancement suggestive for residual/recurrent disease in the ablated zone at the site of the ^111^In-girentuximab uptake after treatment. During a mean FU of 21 months (range 1–33) no other cases with residual/recurrent disease were detected.

**Conclusion:**

FU imaging with ^111^In-girentuximab-SPECT is feasible after ccRCC cryoablation and may contribute to early detection of residual or recurrent disease.

## Introduction

The detection of residual or recurrent disease after ablative therapy of renal tumors on follow-up (FU) cross-sectional imaging is challenging [1]. The specificity to detect vital tumor after ablative therapy based on contrast enhancement or lesion size is limited [2]. This difficulty may be overcome by targeted imaging harboring a superior diagnostic accuracy. To date, no studies evaluating the feasibility of targeted imaging modalities following ablative therapies for renal tumors are available.

Of all renal malignancies, 75% are clear cell renal cell carcinomas (ccRCC) [3]. Because of the high and specific expression levels of carbonic anhydrase IX (CAIX) in 95% of ccRCC, radiolabeled anti-CAIX chimeric monoclonal antibody girentuximab can be used for the detection of ccRCC [4, 5]. Previous studies have shown high accuracy in the detection of ccRCC using single photon emission computed tomography (SPECT) with girentuximab radiolabeled with ^111^Indium (^111^In) [6].

The objective of this study was to evaluate the feasibility of targeted FU imaging 1 month after magnetic resonance (MR)-guided cryoablation using SPECT after ^111^In-labeled girentuximab administration.

## Materials and methods

### Patient selection

In this prospective study, approved by the Regional Internal Review Board (clinicaltrials.gov; NCT 02411968), 16 patients who were planned to undergo percutaneous cryoablation of a T1a (≤ 4 cm) renal tumor were consecutively recruited between February 2015 and September 2018. Inclusion criteria were at least one untreated T1a tumor in one kidney, > 50 years of age, and signed informed consent form.

### Girentuximab conjugation and radiolabeling

Girentuximab (Wilex AG, Munich, Germany) was conjugated with 1,4,7,10-tetraazacyclododecanetetraacetic acid (DOTA, Macrocyclics, Dallas, TX, USA) as described before [7]. Prior to administration the girentuximab-DOTA conjugate was labeled with ^111^In (Mallinckrodt Pharmaceuticals, ‘s Hertogenbosch, The Netherlands). Radiochemical purity was determined by instant thin-layer chromatography and exceeded 95% for all preparations. CAIX-specific binding of the radio-labeled girentuximab was confirmed by determination of the immunoreactive fraction as described by Lindmo et al. and exceeded 70% for all preparations [8].

### Preoperative imaging

To assess tumor targeting before treatment, SPECT/CT (Symbia T16 Truepoint SPECT/CT scanner, Siemens Healthcare, The Hague, The Netherlands) of the abdomen was performed 4 to 5 days after injection of ^111^In-girentuximab (10 mg, 100 MBq). Because local disease status was evaluated, no whole body scans were performed. Patients were positioned supine with arms lowered alongside the body. After acquisition of a low-dose, non-contrast-enhanced CT, SPECT images were acquired with a 15% window around 172 and 247 keV peaks using medium-energy parallel-hole collimators. Images were obtained clockwise with a dual-headed camera, and continuously from 0 to 180 °, with 64 views, 19 s per view (total of 128 views in 360°). The matrix size was 128*128. Transverse and coronal SPECT images were reconstructed (iterative reconstruction, flash 3D, 6 iterations, 16 subsets); attenuation correction of the SPECT was performed using the non-contrast CT images. Scatter correction was performed together with the attenuation correction.

Renal tumors were scored positive or negative based on an arbitrary visual evaluation by a nuclear physician blinded to the FU results (WO, 24 years of experience in nuclear medicine). A positive lesion was defined as a renal lesion with ^111^In-girentuximab uptake corresponding with the lesion suspect for RCC detected at preoperative cross-sectional imaging. All images were reviewed on a picture archiving and communication system (PACS; IMPAX, Agfa Healthcare, Mortsel, Belgium) with additional review using dedicated software for molecular imaging if deemed necessary (Hermes Medical Solutions, Stockholm, Sweden).

### Follow-up imaging

In case of a positive preoperative scan, FU SPECT was obtained 1 month after cryoablation 4 to 5 days after injection of ^111^In-girentuximab. The presence of residual or recurrent disease at the site of the ablated lesion was assessed similar to the scan at baseline. Because the treatment was performed under MRI guidance, the regular FU consisted of MRI scans at 1, 3, 6, 12, and 18 months FU and annually thereafter. FU MR scans were acquired using a 3 T MRI system (Magnetom Trio, Siemens, Erlangen, Germany). The scanning protocol consisted of anatomical T1-weighted VIBE (volumetric interpolated breath-hold examination), with and without fat suppression, and T2-weighted fat-suppressed HASTE (Half-Fourier-Acquired Single-shot Turbo spin Echo) sequences in axial and coronal directions using breath hold. Water diffusion was measured in three directions using a respiratory-triggered coronal single-shot echo-planar imaging (SS-EPI) sequence with b-values of 50, 800, and 1400 s/mm^2^. Subsequent dynamic contrast-enhanced (DCE) fat-saturated T1-weighted VIBE 3D imaging was acquired in the corticomedullary, nephrographic, and excretory phase. In case of suspicion for metastatic disease or intolerance for gadolinium contrast agent CT scans (Aquilion one, Canon Medical Systems, Zoetermeer, The Netherlands) were obtained during FU. CT scans were acquired after iodine-based contrast administration in at least the porto-venous or corticomedullary phase. The presence or absence of vital tumor tissue was based on FU CT and MRI findings reviewed by a radiologist (JF, 15 years of experience in genitourinary imaging). Persistent or newly diagnosed nodular contrast enhancement at the site of the ablated lesion and growth of the ablated lesion in time were considered suggestive for vital tumor tissue [2].

### Statistics

For statistical analysis SPSS (version 22.0; IBM; Amonk; New York) was used. Continuous variables are expressed as mean (range).

## Results

**Fig. 1:**
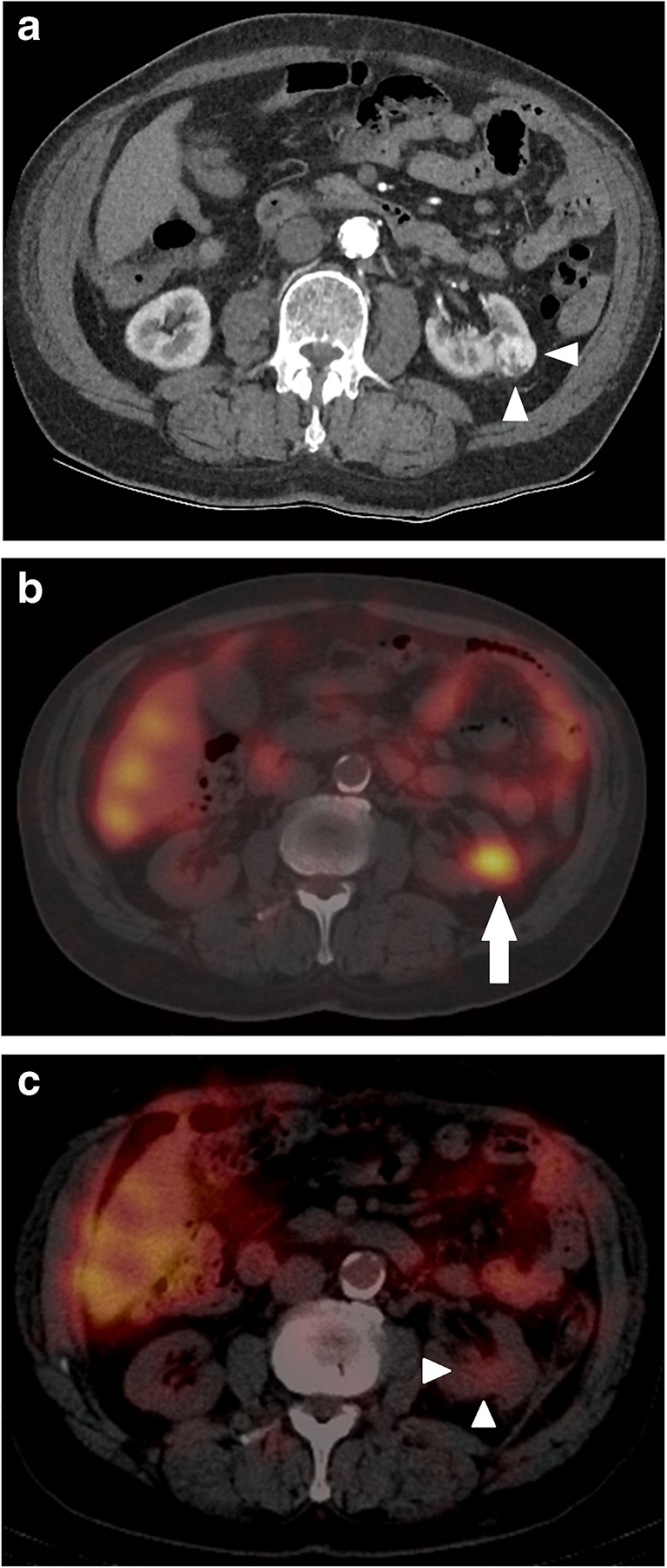
a. In this 70-year-old male patient, an incidentaloma was found in the left kidney on ultrasound. This axial contrast enhanced CT image in the corticomedullary phase confirms the presence of a 25 mm process suspicious for malignancy (white arrowheads). Biopsy confirmed clear cell renal cell carcinoma. b. Axial SPECT image showed uptake of the lesion (white arrow) before cryoablation was performed. c. One month after percutaneous cryoablation SPECT showed no uptake of the ablated lesion. Very low uptake was seen surrounding the ablated lesion (white arrowheads)

**Fig. 2:**
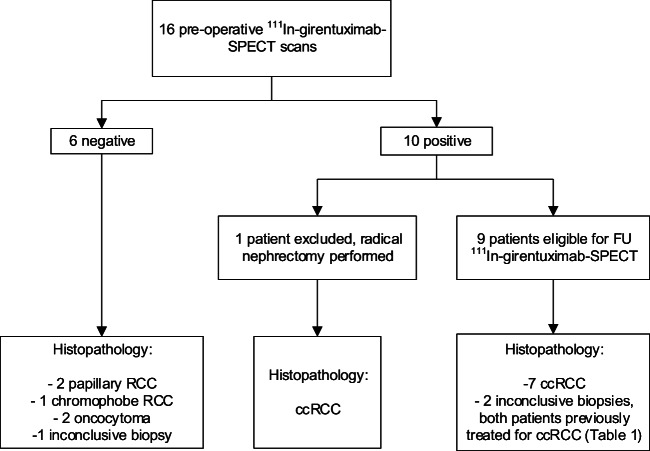
Patient flowchart. *SPECT* single photon emission computed tomography; *FU* follow-up; *ccRCC* clear cell renal cell carcinoma

Sixteen patients underwent an ^111^In-girentuxumab SPECT before renal cryoablation: 10 (63%) of these patients showed positive targeting of the lesion with evident tumor-to-normal ratios (Fig. 1). Patient flow and histological findings in case of negative SPECT are provided in Fig. 2. In one patient, the ^111^In-girentuxumab SPECT showed new findings, making the lesion unsuitable for treatment with cryoablation. Nine patients with a positive SPECT scan were eligible to undergo FU SPECT imaging. Four patients were female (44%), mean age was 73.9 years (range 58–83 years), and mean tumor diameter was 28 mm (range 12–44 mm) (Table 1).

**Table 1:** Demographics, patient, and tumor characteristics of patients eligible for follow-up SPECT

Patient	Age (years)	Sex	Tumor diameter (mm)	1 month FU ^111^In-girentuximab-SPECT	MRI FU imaging at 1 month	FU imaging during further FU (modality)	FU duration (months)	Biopsy before ablation	Remarks
1	79	F	24	Negative	Negative	Negative (CT)	33	ccRCC	Allergic reaction on gadolinium-based contrast
2	71	F	23	Negative (high non-specific uptake outside ablated lesion)	Negative	Negative (MRI)	16	cc RCC	On hemodialysis due to diabetic nephropathy.
3	70	M	25	Negative	Negative	Negative (MRI)	1	ccRCC	Died 2 months after last FU imaging due to cause unrelated to RCC
4	82	F	30	Negative	Negative	Negative (MRI)	32	ccRCC	
5	72	M	28	Negative	Negative	Negative (MRI)	33	Inconclusive	Previous contralateral nephrectomy and ipsilateral cryoablation for ccRCC
6	71	F	12	Negative	Negative	Negative (MRI)	30	Inconclusive	Previous radical nephrectomy and ipsilateral partial nephrectomy for ccRCC
7	79	M	32	Positive	Negative	Positive at 6 months FU (CT)	14	Not performed	Initial treatment for local control primary tumor after radiotherapy of single synchronous osseous metastasis (biopsy proven ccRCC). Died after 15 months FU due to progressive disease. Treated with sunitinib after treatment primary tumor
8	83	M	44	Negative	Negative	Negative (MRI)	17	ccRCC	
9	58	M	30	Negative	Negative	Negative (CT)	15	ccRCC	Previous contralateral radical nephrectomy for ccRCC, known metastatic disease, Von Hippel Lindau, targeting for girentuximab proven on ^89^Zr-girentuximab SPECT^7^

*FU* follow-up; *SPECT* single photon emission computed tomography, *ccRCC* clear cell renal cell carcinoma

**Fig. 3:**
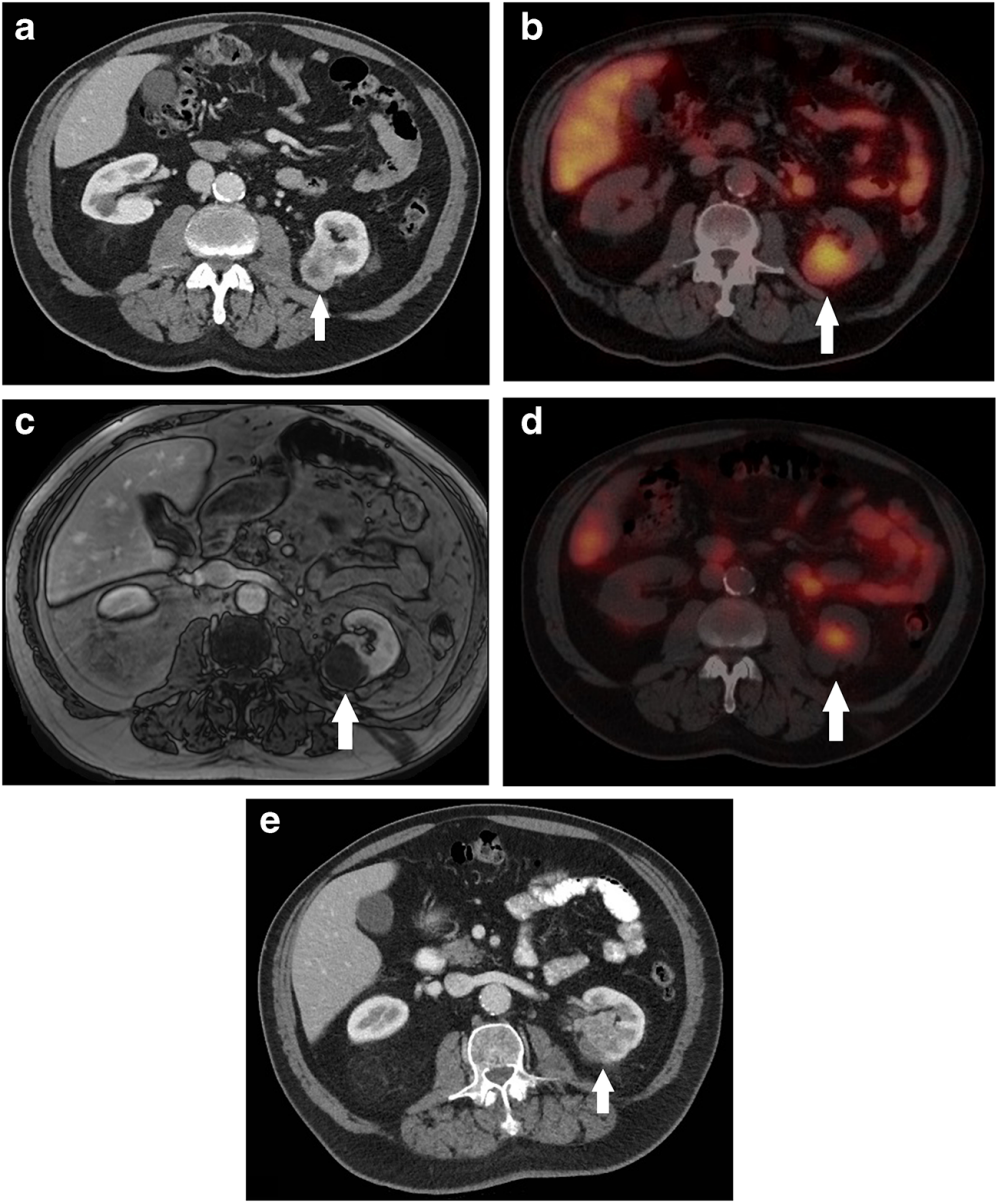
a. In this 79-year-old patient with one known osseous metastasis proven to be clear cell renal cell carcinoma on biopsy, the 32-mm-large primary lesion, as seen on this contrast enhanced axial CT image in the corticomedullary phase (white arrow), was treated with cryoablation. b. The axial preoperative SPECT shows uptake of the tumor (white arrow). c. Axial follow-up MR image at 1 month FU (dynamic contrast-enhanced fat-saturated T1-weighted VIBE sequence) showed no contrast enhancement of the ablated lesion in the corticomedullary phase suggestive for vital tumor presence. d. One month follow-up axial SPECT image showed uptake at the ventral/medial site of the ablated lesion suggestive for residual disease (white arrow). e. Axial 6-month follow-up contrast-enhanced CT image in corticomedullary phase shows nodular contrast enhancement centrally located in the left kidney at the site of the previous ^111^In-girentuximab uptake suggestive for residual/recurrent disease (white arrow)

In one patient FU, ^111^In-girentuximab SPECT showed uptake in the ablated lesion, which was suggestive for residual vital tumor. In contrast, the 1 month FU MRI showed no contrast enhancement in the ablated lesion suggestive for residual disease. A CT scan of the thorax and abdomen was obtained 6 months after treatment and showed contrast enhancement in the ablated lesion at the site of the previous ^111^In-girentuximab uptake suggestive for the presence of vital tumor (Fig. 3). Furthermore, the CT showed minimal progression of the known bone and lung metastasis. The patient eventually died of disease progression after 15 months FU (Table 1).

Eight FU ^111^In-girentuximab SPECT scans were scored negative. No residual or recurrent disease was found during CT/MRI FU imaging with a mean FU of 21 months. Follow-up ranged from 14 to 33 months in 8 patients, one patient died 2 months after 1 month FU imaging due to an unrelated cause (Table 1). The negative scans showed low uptake compared to initial tumor uptake surrounding the ablated lesion (Fig. 1).

## Discussion

Due to the high and specific expression levels of the CAIX antigen in clear cell RCC, targeted imaging using the anti-CAIX antibody girentuximab labeled with ^111^Indium can overcome the issue of inconclusive findings on contrast-enhanced cross-sectional FU imaging findings after cryoablation. We showed that early detection of residual disease is feasible.

It is well established that CAIX expression is related to hypoxia in non-ccRCC tissues [9]. Therefore, some level of ^111^In-girentuximab uptake was expected in the area surrounding the ablated lesion during follow-up. In this study, only very moderate levels of uptake and to a far lesser extend compared to the initial tumor were seen in the renal parenchyma surrounding the ablated lesion. This enabled correct evaluation of the FU SPECT scans in terms of vital tumor presence.

Compared to SPECT, better contrast and spatial resolution can be obtained by using a positron emission tomography (PET) tracer. The first study evaluating the use of ^89^Zr-girentuximab-PET/CT in diagnostic setting for ccRCC was published last year and confirmed high diagnostic accuracy for ccRCC [5]. Compared to SPECT, different radiolabels but the same molecular target (CAIX) and antibody (girentuximab) are used for targeted PET imaging of ccRCC. Therefore, PET imaging with radiolabeled girentuximab is, likewise to SPECT, expected to be feasible for FU after cryoablation and should be the focus of future studies because it may increase diagnostic accuracy. At time of the study initiation, ^89^Zr-labeled girentuximab for immunoPET was not available at our institution. Therefore, despite the inferior imaging performance, ^111^In-girentuximab was used as a tracer in this study.

Early detection of residual disease after renal cryoablation is beneficial for several reasons. First, after incomplete RCC ablation increased proliferation of residual tumor cells has been described in a translational murine model, which may result in aggressive local regrowth [10]. Early salvage treatment can avoid local problems due to these phenomena. Second, contrast enhancement of the ablated lesion is seen in up to 50% of the cases on CT and MRI within the first 3 months after treatment, but in only a small part of these cases, the suspicion for residual disease persist during follow-up, and salvage treatment is indicated [11]. Despite the relatively low chance of residual disease, presence of contrast enhancement during early follow-up warrants intensive follow-up with frequent imaging. Our results suggest that early follow-up imaging with ^111^In-girentuximab SPECT may reliably exclude the presence of residual disease, avoiding the necessity for intensified and frequent follow-up imaging in a large group of patients.

To make this imaging modality eligible as a routine follow-up imaging modality, its superior diagnostic accuracy over conventional contrast-enhanced cross-sectional imaging should be proven. Also, the availability of this imaging modality is still limited hampering routine use. At this point, ^111^In-girentuximab SPECT may be especially useful in case of inconclusive CT or MRI findings or in case incomplete tumor ablation is expected and residual disease must be detected in an early stage.

This study has several limitations. Due to the design of this feasibility study, only a small number of patients were included. Although the feasibility of ^111^In-labeled girentuximab SPECT appeared evaluable, the diagnostic accuracy of this targeted imaging modality can only be assessed in a larger patient cohort. Also, most likely due to the limited number of included patients, no false positive or negative scans occurred in this study, which could lead to possible overestimation of the expected diagnostic accuracy. Only when observing false negative or positive results, the limitations of ^111^In-girentuximab SPECT in this setting can be evaluated. Future studies in this field should preferably focus on using a PET tracer. This study will be initiated. In conclusion, ^111^In-girentuximab SPECT is a feasible FU imaging modality after ccRCC cryoablation and may aid in the early detection of residual or recurrent ccRCC. It can overcome the issue of inconclusive findings on contrast-enhanced cross-sectional FU imaging.
